# Nucleoprotein of a Rice Rhabdovirus Serves as the Effector to Attenuate Hemolymph Melanization and Facilitate Viral Persistent Propagation in its Leafhopper Vector

**DOI:** 10.3389/fimmu.2022.904244

**Published:** 2022-05-17

**Authors:** Ruonan Zhang, Xiao-Feng Zhang, Yunhua Chi, Yuanyuan Xu, Hongyan Chen, Zhongxin Guo, Taiyun Wei

**Affiliations:** State Key Laboratory of Ecological Pest Control for Fujian and Taiwan Crops, Institute of Plant Virology, Fujian Agriculture and Forestry University, Fuzhou, China

**Keywords:** rice stripe mosaic virus, melanization, leafhopper, prophenoloxidases (PPO), viral infection

## Abstract

Melanization in the hemolymph of arthropods is a conserved defense strategy against infection by invading pathogens. Numerous plant viruses are persistently transmitted by insect vectors, and must overcome hemolymph melanization. Here, we determine that the plant rhabdovirus rice stripe mosaic virus (RSMV) has evolved to evade the antiviral melanization response in the hemolymph in leafhopepr vectors. After virions enter vector hemolymph cells, viral nucleoprotein N is initially synthesized and directly interacts with prophenoloxidase (PPO), a core component of the melanization pathway and this process strongly activates the expression of PPO. Furthermore, such interaction could effectively inhibit the proteolytic cleavage of the zymogen PPO to active phenoloxidase (PO), finally suppressing hemolymph melanization. The knockdown of PPO expression or treatment with the PO inhibitor also suppresses hemolymph melanization and causes viral excessive accumulation, finally causing a high insect mortality rate. Consistent with this function, microinjection of N into leafhopper vectors attenuates melanization and promotes viral infection. These findings demonstrate that RSMV N serves as the effector to attenuate hemolymph melanization and facilitate viral persistent propagation in its insect vector. Our findings provide the insights in the understanding of ongoing arms race of insect immunity defense and viral counter-defense.

## Introduction

Insect-borne plant viral diseases are widespread and seriously threaten the stability of agricultural production on a global scale. Many economically important plant viruses are transmitted by sap-sucking insects in a persistent manner (1). Therefore, the high-efficiency transmission of plant viruses in insect vectors is crucial for the spread of viruses in the field. Once acquired from the plant phloem, these viruses initially infect the insect intestines, disseminate to the hemolymph, and enter the salivary glands, where they are eventually released to healthy plants (2, 3). During this process, the virus must encounter various types of antiviral immune pathways in the vector, including the small interfering RNA antiviral pathway, melanization, autophagy, apoptosis, and stress-regulated signaling pathways (4–7). Growing evidence suggests that plant viruses have evolved various counter-strategies to impair immune defense or even exploit these pathways to promote infection (8).

How viruses evade the immune antiviral pathway in the hemolymph of insect vectors is a prerequisite for viral transmission. For example, the cuticular protein can effectively stabilize the tenuivirus rice stripe virus (RSV) particles in the hemolymph of the planthopper vector (9). The melanization response is an essential defense mechanism that specifically occurs in the insect hemolymph (10–12). Melanin, the final product of the melanization reaction, plays a vital role in defending insects against pathogenic bacteria, fungi, viruses, and parasitic wasps (13). Once pathogen-associated molecular patterns are recognized, the prophenoloxidase (PPO) is converted to active phenoloxidase (PO) *via* a serine protease cascade (14–17). With the participation of molecular oxygen, PO catalyzes the conversion of phenols to quinones and mediates the formation of melanin, which directly encapsulates and kills certain pathogens or parasites (18–22). Different viruses have developed the relative mechanisms to suppress melanization (23–28). For example, RSV ensures viral stability in the hemolymph of the planthopper vector by inhibiting the cleavage ability of PPO (23). Thus, plant viruses potentially evolved the conserved counter-strategies to impair the melanization response in the hemolymph to promote viral persistence of insect vectors.

Rhabdoviruses form a large family whose collective host range includes vertebrates, invertebrates, and plants, and are of considerable socioeconomic and agricultural importance (29). Plant rhabdoviruses that are transmitted by insect vectors in a persistent-propagative manner cause substantial agricultural losses (30). Rice stripe mosaic virus (RSMV), the only rice cytorhabdovirus, was first identified in Guangdong Province, China in 2015 and has posed a serious threat to local rice production in recent years (31). The genome of RSMV, comprising a single-stranded negative RNA, encodes seven canonical proteins in the order 3′-N-P-P3-M-G-P6-L-5′: nucleocapsid protein (N), phosphoprotein (P), nonstructural protein P3, matrix protein (M), glycoprotein (G), nonstructural protein P6, and large polymerase protein (L). Like the N proteins of other rhabdoviruses, RSMV N is a core protein that functions during assembly of the viroplasm and is responsible for viral replication (29, 30). In the field, RSMV is transmitted by the leafhoppers *Recilia dorsalis* and *Nephotettix virescens* in a persistent-propagative manner (31). We previously elucidated the transmission route of RSMV in *R. dorsalis* (32, 33). Once ingested by the leafhopper vector, RSMV passes through the midgut barrier and infects the hemolymph before invading the salivary glands (2). Therefore, the survival from immune defense in vector hemolymph is vital for the systemic dissemination of RSMV. However, how RSMV evades or interferes with melanization in vector hemolymph remains unknown.

In this study, we used the RMSV/*R. dorsalis* infection system to explore the trade-off mechanism between melanization in vector hemolymph and the persistent infection of plant rhabodviruses. We determine that RSMV N suppresses PO-mediated melanization by interfering with the proteolytic cleavage of PPOs. Thus, RSMV N serves as the effector to attenuate hemolymph melanization and facilitate viral persistent propagation in insect vectors. Our findings reveal that plant rhabdoviruses have evolved to evade the antiviral melanization response in vector hemolymph.

## Materials and Methods

### Insect Rearing and Virus Collection

*R. dorsalis* individuals were obtained from Guangdong province, China and reared on fresh rice seedlings in clean plastic containers at 25 ± 2°C, 70 ± 5% relative humidity under a 16 h light/8 h dark photoperiod. RSMV-infected rice plants were collected from rice fields in Luoding, Guangdong province, China and maintained on rice plants *via* transmission by *R. dorsalis* (3).

### Obtaining RSMV Crude Extracts and Microinjection of *R. dorsalis*

Rice leaf tissue (10 g) with RSMV was frozen in liquid nitrogen and quickly ground into a powder with a mortar and pestle. PBS buffer was added at a ratio of 3-5 ml of PBS (pH=8.0) per 10 g rice leaves. The mixture was centrifuged and the supernatant was collected to store for subsequent experiments. To inject RSMV crude extract into *R. dorsalis*, 3-4 instar nymphs were stunned in carbon dioxide and injected with 25 nl of virus solution per nymph. The same volume of crude extracts from healthy rice tissues was injected as a negative control. At 7 d after injection, total RNA was extracted from each group of insects by the Trizol method. RT-qPCR assay was used to detect the virulence rate.

### RT-qPCR Assay

RT-qPCR assay was used to quantify the relative transcript levels of *RSMV*, *PPO*, and various genes in insects. The primers of each gene were designed according to the target gene sequence in NCBI. RT-qPCR assay was performed using a SYBR Green PCR Master Mix kit (Promega, Madison, WI, USA). The thermal cycling conditions were 95°C for 3 min followed by 40 cycles of 95°C for 30 s, 57°C for 25 s, and 70°C for 40 s. *Elongation factor 1* (*EF1*) was used as an internal reference gene. Three biological repeats were used for RT-qPCR assay. The PCR efficiency for each primer couple have been evaluated by SATqPCR (http://satqpcr.sophia.inra.fr/). The relative transcript level of each gene is reported as the mean ± SE. Differences were statistically evaluated using the Student’s t-test in GraphPad Prism 8.0.2.

### Hemolymph Extract Collection

Hemolymph extracts were collected from 50 adult insects per treatment. *R. dorsalis* adults were chilled on ice for 10 min prior to hemocyte extraction. Under a dissecting microscope, a hind leg was detached from the insect body using surgical scissors. 10 µl PBS was then injected into the abdomen of the insect through the wound sites and gently sucked out using a 10 µl pipette. This process was repeated three times. The diluted hemolymph was collected and subjected to the following experiments.

### Electron Microscopy

The hemolymph samples of each group of at least 50 *R. dorsalis* adults infected with RSMV, *Micrococcus luteus*, or the mixture of *M. luteus* and RSMV, were extracted and subjected to phosphotungstic acid (PTA) negative staining. A 10 µl diluted hemolymph extracted samples were placed onto paraffin film, covered with carbon-free aromatic film copper mesh for 10 minutes, and incubated in 10 µl PTA (2%) for 10 seconds. The copper mesh was placed under a baking lamp for 3 minutes, dried, and observed under a transmission electron microscope (Hitachi H-7650).

### *In vitro* Spontaneous Melanization Assay

Hemolymph samples were extracted from *R. dorsalis* adults infected with RSMV, *M. luteus*, *M. luteus*/RSMV at 7 d post first access to the diseased plants (padp). The 10 μl hemolymph extracted samples from each group were mixed with 10 µl of PBS and 20 µl of dopamine (1 mM) and spontaneously oxidized at room temperature. Photographs showing the melanization of the samples were taken every hour.

### PO Activity Assay

To analyze PO activity in hemolymph, a group of 20 viruliferous or nonviruliferous *R. dorsalis* adults were centrifuged at 12,000 rpm/min at 4°C for 15 min. A 30 µl aliquot of supernatant was gently mixed with 100 µl of 1 mM dopamine in 10 mM Tris-HCl buffer (pH 8.0) in a 96-well plate at room temperature for 5 min. The absorbance of melanin was measured every 10 min at 490 nm (A490) using a microplate reader (Tecan, Männedorf, Switzerland). Enzyme activity was calculated using the formula described in the phenoloxidase kit (Geruisi, G0146W) according to the manufacturer’s protocol. Activity assays were independently repeated using hemolymph samples from three biological replicates. PO activity in each group is represented as means ± standard error (SE). Differences were statistically evaluated using the Student’s t-test in GraphPad Prism 8.0.2.

### Knocking Down of *PPO* Expression

A 500-bp fragment of *PPO* or *GFP* was amplified and used as a template for the synthesis of dsRNAs (dsPPO and dsGFP) were synthesized *in vitro* using the T7 RiboMAX Express RNAi System (Promega) according to the manufacturer’s instructions. A 25 nl/per insect of dsPPO or dsGFP (1000 ng/µl) was microinjected into 150 viruliferous or nonviruliferous *R. dorsalis* adults. The microinjected leafhoppers were reared on healthy rice seedlings. At 7 d after microinjection, total RNA was isolated from different treatments and subjected to RT-qPCR detection to assess the relative transcript levels of *PPO*. Three biological repeats were used for RT-qPCR assay and analyzed by Student’s t-test. The accumulation of PPO after microinjection with dsPPO was analyzed by immunoblotting with specific antibody against PPO. *R. dorsalis actin* was used as an internal control.

### Mortality Determination

Fifty treated nymphs in a test tube were individually fed with rice seedlings with the roots wrapped in the absorbent paper. New experimental samples were prepared when the insects showed no signs of survival within the first ten minutes of placement into the tubes due to human error. The number of surviving insects was recorded continuously for 12 d, and an observation record was produced every 48 h. Mortality determination was independently repeated in three biological replicates. Differences were statistically evaluated with GraphPad Prism 8.0.2

### Yeast Two-Hybrid Screening and Verification of Interacting Genes

The full-length ORFs of RSMV *N*, *P*, *M*, *G, L*, and *PPO* were amplified by RT-PCR and cloned to the bait vector pGBKT7 and the prey vector pGADT7 by homologous recombination. The plasmids were co-transformed into competent yeast strain AH109. Yeast cells co-transformed with pGBKT7-53 and pGADT7-T, pGBKT7-lam and pGADT7-T, pGADT7 and pGBKT7-N, as well as pGADT7-PPO and pGBKT7. The yeast was grown on minimal selection medium (SD-Trp-Leu) without leucine and tryptophan at 30°C for 72 h. A single colony was picked and streaked onto minimal selection medium containing X-α-gal without adenine, histidine, leucine, or tryptophan (SD -Ade -His -Leu -Trp) at 30°C for 24 h.

### Expression and Purification of PPO and N Proteins

The full-length ORFs of *PPO* and *N* were cloned to the PET-28b-His and pGEX-4T-GST vectors, respectively, by homologous recombination (34). The recombinant plasmids expressing PPO-His or N-GST were transferred into *E. coli* strain BL21. Following induction with 0.5 mM IPTG overnight at 37°C until the OD600 of the bacterial solution reached 0.5–0.7, the *E. coli* cells were collected by centrifugation and sonicated for 30 min in ice water. The supernatant was collected and loaded onto a 4 ml Ni-NTA agarose column for PPO-His or a 6 ml Glutathione Sepharose column for N-GST. The columns were washed with 10 mM imidazole respectively, and each eluate was collected into a different centrifuge tube. Purified proteins were stored in 20 mM Tris-HCl (pH 7.5) at –80°C.

### GST-Pull Down Assay

Recombinant N-GST proteins purified as described above were incubated with 200 µl Glutathione Sepharose 4B beads at 4°C for 3 h. The beads were centrifuged for 5 min at 500 g and the supernatant was discarded. The beads were rinsed with PBS buffer to remove unbound proteins and incubated with purified PPO-His for 4 h at 4°C. After being centrifuged and washed with elution buffer (300 mM NaCl, 10 mM Na_2_HPO_3_, 3 mM KCl, and 2 M KH_2_PO_4_), the bead-bound proteins were subjected to immunoblotting with GST-tag and His-tag antibodies (Abcam, UK).

### Immunofluorescence Staining Of PPO in *R. dorsalis*

To examine the distribution of PPO in *R. dorsalis*, 3rd instar nymphs from nonviruliferous populations of *R. dorsalis* (n = 20, three biological repetitions) were dissected (34). The digestive systems and hemolymph were fixed in 4% paraformaldehyde at room temperature for at least 8 h, permeabilized in 4% Triton X-100 for 24 h, and immunolabeled with PPO-specific antibody conjugated to FITC (PPO-FITC). The samples were examined under a Leica TCS SP5II confocal microscope. To study the localization of PPO and RSMV N in the hemolymph of RSMV-infected *R. dorsalis*, the hemolymph samples were collected from RSMV-infected adult insects and treated in exactly the same manner with N antibody conjugated to rhodamine (N-rhodamine) and PPO-FITC. The samples were then examined by immunofluorescence microscopy.

### PPO *In Vitro* Cleavage Experiments

To test the effect of N protein on PPO cleavage, a mixture of PPO-His, mPPO-His, hemolymph crude extract, N-GST, and PBS was incubated at 30°C for 1 h. To evaluate the cleavage of PPO, the reaction mixture and controls lacking N-GST were analyzed by immunoblotting using antibody against His-tag. To assay the activity of PO cleaved by hemolymph, the same reaction mixture and controls were analyzed as described in the PO activity assay section.

## Statistical analysis

All quantitative data presented in the text and figures were analyzed by two-tailed *t*-tests with GraphPad Prism 8.0.2 (GraphPad Software, San Diego, CA, USA).

## Results

### RSMV N Interacts With a Homolog of PPO of *R. Dorsalis*

**Figure 1:**
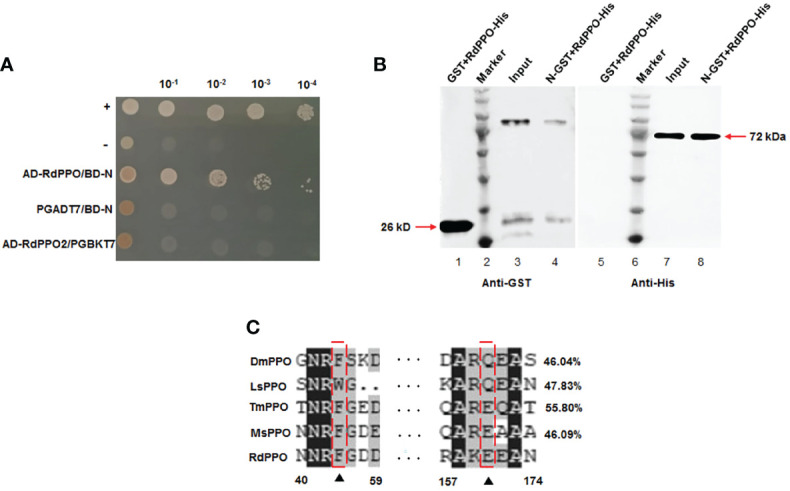
Interaction and co-localization of RSMV N with RdPPO. **(A)** Yeast two-hybrid assay of the interaction between RdPPO and RSMV N. Transformants on SD/-Trp-Leu-Ade-His plates are labeled as follows: +, positive control, pGBKT7-53/pGADT7-T; –, negative control, pGBKT7-Lam/pGADT7-T. Serially diluted yeast cultures are shown. **(B)** Pull-down assay of the interaction of PPO with N. N was fused with GST as a bait protein. PPO was fused with His as a prey protein. Glutathione beads loaded with purified GST protein (lane 1, lane 5) or GST-fused N (GST-N) (lane 3, lane 7) were incubated with recombinant RdPPO-His protein. Following incubation, the amounts of GST-N and RdPPO-His in the reaction mixtures (input) and those bound to the beads (GST pull-down) were determined by immunoblotting using anti-GST and anti-His antibodies. **(C)** Alignment of the conserved sequences of PPO from five insect species. Possible proteolytic cleavage sites are marked with arrows. All conserved residues are highlighted in black. Alignment of the conserved amino acid sequences from GenBank using DNAMAN shows DmPPO1 (NP_476812.1), LsPPO1 (RZF43154.1), MsPPO (L42556.1), and TmPPO (BAA75470.1).

We previously demonstrated that RSMV N contributed to the assembly of the viroplasm and was essential for viral replication (2). We then used RMSV N as a bait protein to screen its interacting insect factors from a yeast cDNA library of *R. dorsalis*. Sequencing of the positive clones identified the N-terminal region (384 aa) of *R. dorsalis* PPO (RdPPO). We cloned the full-length ORF of *PPO* gene (1851 bp) based on analysis of *R. dorsalis* RNA-seq data. We demonstrated that the interaction between RdPPO and RSMV N by performing a yeast two-hybrid assay (**Figure 1A**). A glutathione S-transferase (GST) pull-down experiment confirmed the interaction of RdPPO and RSMV N (**Figure 1B**). Sequence alignment indicated that RdPPO shared 55.80%, 46.09%, 46.04%, and 47.83% identify with *Tenebrio molitor* PPO (TmPPO), *Manduca sexta* PPO (MsPPO), *Drosophila melanogaster* PPO (DmPPO), and *Laodelphgax striatellus* PPO (LsPPO), respectively. Two candidate cleavage sites were identified in these PPO proteins (**Figure 1C**). Bioinformatics analysis indicated that PPOs are evolutionarily conserved among different insects, suggesting that these proteins may also share the similar functions in the melanization response.

### RdPPO Manipulates Melanization in the Hemolymph of Nonvirulifeorus *R. dorsalis*

**Figure 2:**
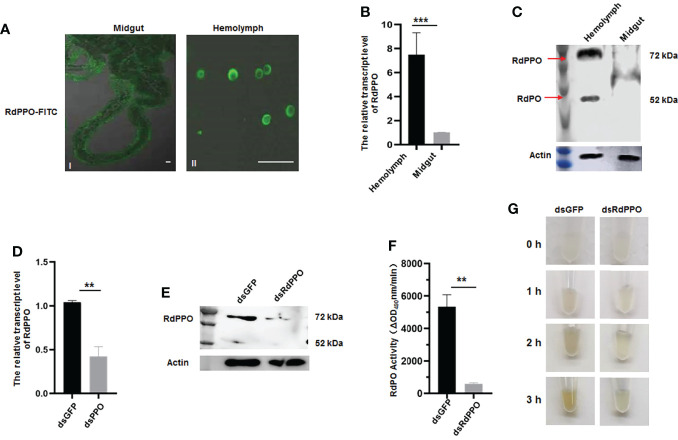
The role of RdPPO in the melanization reaction of nonviruliferous *R. dorsalis.* **(A)** Distribution of RdPPO in the midgut and hemolymph of nonviruliferous *R. dorsalis*. Leafhopper organs and tissues were dissected and analyzed by immunofluorescence assay. The alimentary canals and hemolymph were immunolabeled with RdPPO-specific antibody conjugated to FITC and examined by confocal microscopy. **(B)** Transcript levels of *RdPPO* in the midgut and hemolymph of healthy leafhoppers were normalized to the transcript levels of the internal control *Actin* gene. **(C)** RdPPO protein levels in the midgut and hemolymph of healthy leafhoppers. Total proteins were extracted from the dissected midguts or hemolymph of healthy leafhoppers and subjected to immunoblotting with RdPPO-specific antibody. The bands containing RdPPO and RdPO are labeled with red arrows. Leafhopper Actin was used as an internal control. **(D)** Transcript levels of *RdPPO* genes in *R. dorsalis* after microinjection with dsRNAs (dsGFP or dsRdPPO), as detected by RT-qPCR. The transcript levels of *PPO* were normalized to the transcript levels of the internal control *Actin* gene in whole bodies of nonviruliferous *R. dorsalis* at 7 d post-microinjection of dsPPO and dsGFP. P-values were estimated using Student’s t-test. **(E)** PPO levels in *R. dorsalis* after treatment with dsGFP or dsRdPPO. At 7 d post-microinjection, total protein were extracted from 15 nonviruliferous *R. dorsalis* injected with dsGFP or dsRdPPO and subjected to immunoblotting with RdPPO-specific antibody. The bands containing RdPPO and RdPO are labeled with red arrows. Leafhopper Actin was used as an internal control. **(F)** PO activity in the hemolymph of *Recilia dorsalis* injected with dsGFP or dsRdPPO. Hemolymph samples were collected at 5 d after treatment and subjected to activity assays. Each value is given as the mean ± SEM of three replicates. Asterisks indicate differences (*, P < 0.05) between the indicated groups. **(G)** *In vitro* spontaneous melanization assay of the hemolymph from leafhoppers subjected to different treatments. Hemolymph was collected from *R. dorsalis* injected with dsGFP or dsRdPPO and subjected to a spontaneous melanization assay. The biosynthesis of melanin was recorded by photography at different time points from 0 to 3 **(h)** Scale bars, 20 μm.

To clarify the function of RdPPO, we purified the C-terminus of RdPPO (699 aa) in *E. coli* and prepared a polyclonal antibody by immunizing rabbits. We then investigated the distribution of RdPPO in different organs of healthy *R. dorsalis* by immunofluorescence microscopy with the antibody against RdPPO conjugated with FITC (RdPPO-FITC). No signals of the RdPPO antigen were observed in the intestines of *R. dorsalis*, whereas the strong immunofluorescence signals of RdPPO were detected in hemolymph cells (**Figure 2A**). We then measured the mRNA and protein expression levels of RdPPO in the intestine and hemolymph *via* RT-qPCR and immunoblotting. The results of these experiments were consistent with the results of immunofluorescence microscopy, suggesting that RdPPO was preferentially expressed in the hemolymph cells of *R. dorsalis* compared to other regions (**Figures 2B, C**). Furthermore, the proteolytic cleavage of the zymogen RdPPO to RdPO was observed in the insect hemolymph (**Figure 2C**).

To further verify the role of RdPPO in melanization, we silenced *RdPPO* by microinjecting dsRdPPO into the body of *R. dorsalis* and evaluated the effects of dsRNAs treatment *via* RT-qPCR and immunoblotting. Compared to *R. dorsalis* injected with dsGFP, the mRNA and protein levels of RdPPO were strongly reduced at 7 d after treatment with dsRdPPO (**Figures 2D, E**). Moreover, an immunoblotting assay indicated that the accumulation of RdPO was also reduced after the knockdown of *RdPPO* expression (**Figure 2E**). We then measured PO activity by extracting hemolymph from a group of 20 *R. dorsalis* adults microinjected with either dsRdPPO or dsGFP and subjecting the samples to a PO activity assay. As expected, the knockdown of *PPO* expression caused a decrease in PO activity in the hemolymph of *R. dorsalis* (**Figure 2F**).

Since PO is the final enzyme that catalyzes melanin biosynthesis, we examined the spontaneous melanization of hemolymph samples from dsPPO- or dsGFP-treated *R. dorsalis*. As shown in **Figure 2G**, after 3 h treatment, the melanin was observed in the hemolymph samples from the dsGFP-treated group, whereas only slight melanin accumulation was observed in the dsPPO-injected control. These results suggest that the silencing of *PPO via* dsRNAs microinjection impedes the melanization response in the hemolymph of *R. dorsalis*. Taken together, these results indicate that RdPPO is mainly accumulated in the hemolymph of *R. dorsalis*, and the conversation of RdPPO into active RdPO determines the progression of the melanin immune response.

### The Melanization Plays an Antiviral Role during RSMV Infection in *R. Dorsalis*

**Figure 3:**
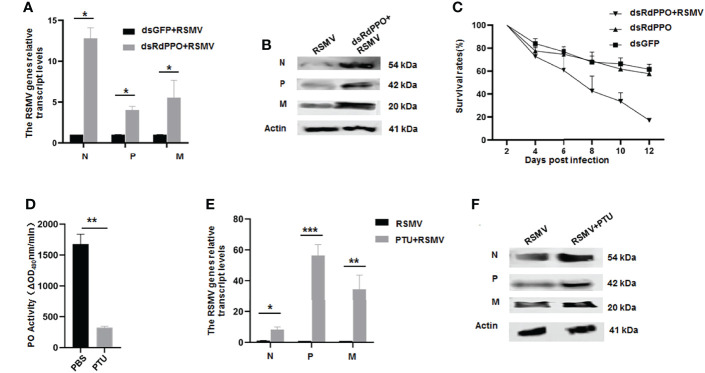
The antiviral function of melanization during RSMV infection of *R. dorsalis*. **(A)** Transcript levels of RSMV genes in viruliferous *Recilia dorsalis* microinjected with dsRNAs analyzed by RT-qPCR. At 7 d post microinjection, RT-qPCR was used to detect the transcript levels of N, P, and M genes in different groups. **(B)** RSMV N, P, and M levels in viruliferous *Recilia dorsalis* at 5 d post-microinjection with dsRdPPO or dsGFP, as detected by immunoblotting with specific antibodies. Insect Actin protein was used as an internal control. **(C)** Survival rates of dsRdPPO- or dsGFP-treated viruliferous and nonviruliferous *R. dorsalis*. Each point represents the mean value of triplicates, and the error bars indicate standard deviation. **(D)** Variations in PO activity in viruliferous *R. dorsalis* after injection of PBS of PTU. Hemolymph samples were collected at 5 d post-infection from at least 20 leafhoppers per group and subjected to activity assays. **(E, F)** Variations in N, P, and M RNA and protein levels in whole bodies of viruliferous *R. dorsalis* after treatment with PTU. The RNA levels of N, P, and M were quantified by RT-qPCR **(E)**. Specific antibodies of N, P, and M were used to quantify protein levels *via* immunoblotting. Insect Actin protein was used as an internal control **(F)**. *P < 0.05; **P < 0.01; ***P < 0.001.

We next investigated the antiviral function of melanization by the knockdown of *RdPPO* expression in viruliferous *R. dorsalis*. We microinjected a mixture of RSMV crude extract with dsRdPPO or dsGFP into the bodies of *R. dorsalis* and evaluated viral infection by RT-qPCR and immunoblotting. At 7 d after microinjection, viral accumulation was dramatically increased after the knockdown of *RdPPO* expression (**Figures 3A, B**), suggesting that the inhibition of melanization promoted RSMV propagation in *R. dorsalis.* At 12 d after microinjection, the dsRdPPO-treated viruliferous insects showed a survival rate of approximately 16% compared to ~62% in the dsGFP-treated controls (**Figure 3C**). We reasoned that the low survival rate of viruliferous *R. dorsalis* with dsPPO treatment might be associated with a considerable increase in viral infection. These results demonstrate that the melanization effectively modulates the persistent infection of RSMV in *R. dorsalis.*

To further validate the antiviral function of melanization during RSMV infection, we impaired the melanization response in *R. dorsalis* using the PO inhibitor phenylthiourea (PTU). We first tested a series of PTU concentrations *via* microinjection into the bodies of *R. dorsalis*. PTU at a concentration of 0.5 mM effectively suppressed PO activity and did not significantly cause the phenotypic abnormalities or the mortality of nonviruliferous *R. dorsalis* (**Figure 3D** and **Supplementary Figure 1**). At 7 d post microinjection of 0.5 mM PTU into viruliferous *R. dorsalis*, viral accumulation was markedly increased compared to the control (**Figures 3E, F**). PTU treatment also led to a higher insect mortality rate (**Supplementary Figure 1**). These results confirmed that virus-induced melanization in vector hemolymph could inhibit RSMV propagation.

### Melanization is Impaired During RSMV Infection in *R. Dorsalis*

**Figure 4:**
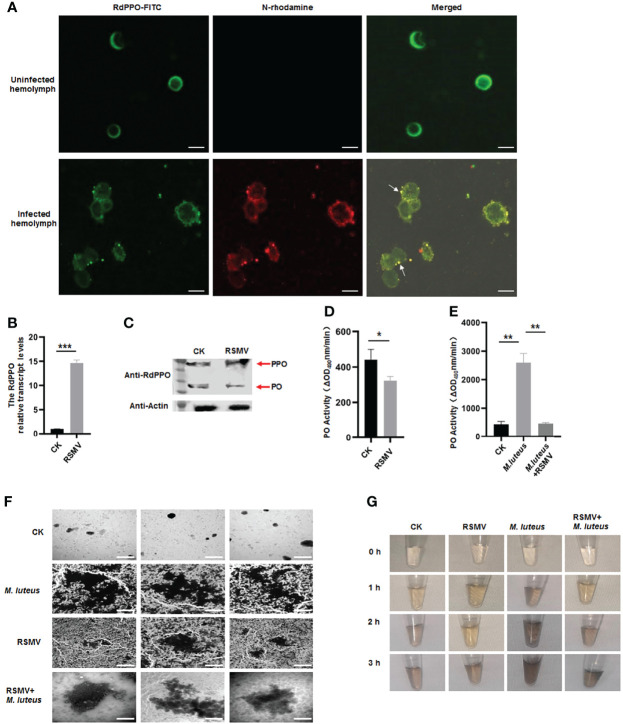
Melanization is inhibited in RSMV infected leafhopper. **(A)** RdPPO and N distribution in hemolymph cells of RSMV-infected *R. dorsalis*. Hemolymph cells collected from viruliferous *R. dorsalis* were subjected to an immunolabeling assay with anti-RdPPO antibody conjugated with FITC (green) and anti-N antibody with rhodamine (red) and observed by confocal microscopy observation. Scale bars, 5 μm. **(B)** Transcript levels of *PPO* in RSMV-infected or healthy *R. dorsalis* detected by RT-qPCR normalized to the transcript levels of the internal control *Actin* gene. **(C)** The abundance of PPO and PO in viruliferous and nonviruliferous *R. dorsalis* detected by immunoblotting. RdPPO-specific antibody was used to detect RdPPO and RdPO proteins. The abundance of *R. dorsalis* Actin, detected with actin antibody, was used as the control. Red arrows indicate the RdPPO and RdPO bands. **(D)** PO activity in the hemolymph of RSMV-infected and nonviruliferous *R. dorsalis*. Adult insects were punctured and their hemolymph examined for PO activity. PO activity was strongly reduced in viruliferous *R. dorsalis* compared to the nonviruliferous controls. **(E)** PO activity in nonviruliferous or viruliferous *Recilia dorsalis* infected with *M. luteus*. The control group was injected with PBS. **(F)** Transmission electron microscopy images showing the varied morphology of melanin produced in hemocytes of leafhoppers infected with *M. luteus*, RSMV, or RSMV/*M. luteus*. Scale bars, 2 µm. **(G)** *In vitro* spontaneous melanization assay of hemolymph from leafhoppers subjected to different treatments. Hemolymph was collected from *R. dorsalis* infected with *M. luteus*, RSMV, or RSMV/*M. luteus* and subjected to an *in vitro* spontaneous melanization assay. Melanin production was recorded by photography at different time points from 0 to 5 h. *P < 0.05; **P < 0.01; ***P < 0.001.

Since RSMV N interacted with RdPPO, we next investigated whether RSMV infection influenced the melanization response in vector hemolymph. Immunofluorescence microscopy confirmed that RSMV N was colocalized with RdPPO in the punctate structures in the hemolymph cells during RSMV infection in *R. dorsalis* (**Figure 4A**). We then examined the association between RdPPO expression and RSMV infection in the hemolymph. RT-qPCR and immunoblotting assays revealed that the expression of RdPPO was significantly increased in the hemolymph of viruliferous *R. dorsali* (**Figures 4B, C**). However, the accumulation of RdPO was extremely reduced, suggesting that the conversion of RdPPO into RdPO was inhibited during RSMV infection. Consistent with the results of immunoblotting, RdPO activity was also reduced in viruliferous *R. dorsalis* (**Figure 4D**). These results demonstrate that RSMV infection impairs hemolymph melanization.

To further verify the effect of RSMV infection on hemolymph melanization, we microinjected *M. luteus*, a Gram-positive bacterium, into nonviruliferous or viruliferous *R. dorsalis* adults. PO activity was strongly increased at 24 h after the microinjection of *M. luteus* into nonviruliferous *R. dorsalis*; however, *M. luteus* infection did not significantly alter RdPO activity in RSMV-infected hemolymph (**Figure 4E**). Thus, the melanization induced by *M. luteus* infection was suppressed by RSMV infection. We then observed the melanin synthesis in the hemolymph samples from *M. luteus*-infected viruliferous or nonviruliferous insects by electron microscopy. More melanin aggregates were observed in the hemolymph of *M. luteus*-infected nonvirulifeorus insects compared to *M. luteus*-infected virulifeorus insects (**Figure 4F**). We then performed a spontaneous melanization assay of these hemolymph samples. Consistent with the electron microscopic observations, at 2 h, abundant melanin was observed in the hemolymph samples from *M. luteus*-infected nonviruliferous insects, whereas only low levels of melanin were detected in the hemolymph samples after coinfection of *M. luteus* and RSMV (**Figure 4G**). Taken together, these results demonstrate that RSMV infection impairs melanization in the hemolymph by preventing the conversation of RdPPO to RdPO.

### RSMV N Suppresses PO Activity to Enhance Viral Infection by Inhibiting the Cleavage of RdPPO

**Figure 5:**
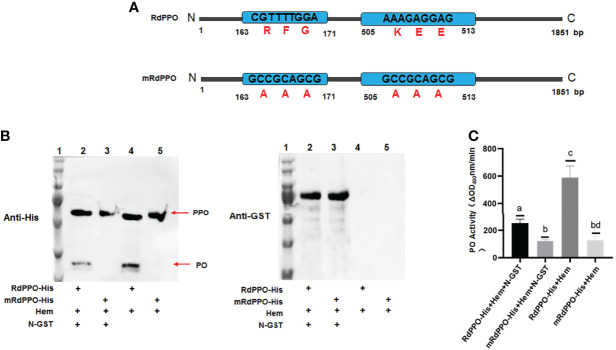
RSMV N suppresses *in vitro* cleavage of RdPPOs into active RdPO. **(A)** Diagrams of RdPPO protein and RdPPO mutant protein (mRdPPO). The amino acids in the cleavage sites of mRdPPO were substituted with alanine, **(A, B)** Purified RdPPO-His (20 ng, 10 μL) or mRdPPO-His (20 μg, 10 μL), N-GST (20 ng, 10 μL), and hemolymph samples (Hem, 20 μL) were incubated at 30°C for 1 (h) The reaction mixture and controls without N-GST were separated by 10% SDS/PAGE and examined by immunoblotting with 6×His antibody (left) or GST antibody (right) (lane 1, position of the protein marker; lane 2, mixture of RdPPO-His/N-GST/Hem; lane 3, mixture of RdmPPO-His/N-GST/Hem; lane 4, mixture of PPO-His/Hem; lane 5, mixture of RdmPPO -His/Hem. The PPO precursor and PO are marked with arrows. **(C)** PO activity of the samples in **(A)**. Mixtures were collected at 1 h post incubation and subjected to PO activity assays. Each value is given as the mean ± SEM of three replicates. Different letters indicate significant difference based on multiple comparisons (Turkey method) after ANOVA.

Since RSMV N interacted with RdPPO, we next investigated whether N repressed the proteolytic cleavage of RdPPO into RdPO. We created RdPPO mutant (mRdPPO) protein in which the amino acid cleavage sites were substituted by alanine (**Figure 5A**). We mixed hemolymph extract from nonviruliferous *R. dorsalis* with purified RdPPO-His and mRdPPO-His with or without N-GST, incubated the samples at 37°C for 1 h, and subjected them to immunoblotting. RdPPO-His, but not mRdPPO-His, was digested to form RdPO *in vitro* by serine protease in the *R. dorsalis* hemolymph extract (**Figure 5B**). Importantly, treatment with N-GST attenuated the cleavage of RdPPO (**Figure 5B**). These results indicate that RdPPO was digested by hemolymph *in vitro*, whereas N specifically prevented this process. We also evaluated RdPO activity in different samples. As expected, lower RdPO activity was detected in the samples containing N-GST/RdPPO-His/hemolymph compared to samples lacking N-GST (**Figure 5C**). Together, these results indicate that N represses RdPO activity by manipulating the cleavage of RdPPO.

**Figure 6:**
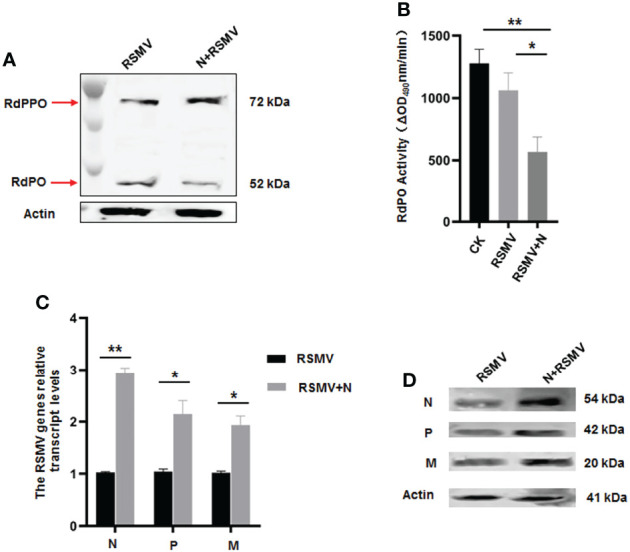
Microinjected purified N protein impairs PO activity and facilitates RSMV infection in *R. dorsalis*. **(A)** PPO and PO levels in viruliferous *R. dorsalis* microinjected with or without purified N determined *via* immunoblotting with PPO-specific antibody. The bands of PPO and PO are indicated. **(B)** Variations in PO activity in viruliferous *R. dorsalis* after injection of purified N. Hemolymph samples were collected at 5 d post injection and subjected to activity assays. **(C)** RT-qPCR showing the transcript levels of RSMV N, P, and M genes in viruliferous *R. dorsalis* after injection of purified N. Results are normalized against the *Actin* expression level. **(D)** Immunoblotting assay showing the levels of N, P and M proteins in viruliferous *R. dorsalis* after injection of purified N. Insect Actin protein was used as an internal control. *P < 0.05; **P < 0.01.

We then microinjected the purified N into viruliferous *R. dorsalis* adults to confirm the functional role of N in repressing RdPO activity *in vivo*. At 6 d after microinjection of purified N, higher RdPPO levels and lower RdPO levels were detected in viruliferous *R. dorsalis* compared to the control (**Figure 6A**), which subsequently led to a decline in RdPO activity in the hemolymph (**Figure 6B**). Consistent with this, a strong increase in RSMV propagation was also detected *via* RT-qPCR and immunoblotting in samples following N microinjection (**Figures 6C, D**). Overall, these results demonstrate that N represses melanization to facilitate RSMV infection by attenuating the conversation of RdPPO to RdPO in *R. dorsalis.*

## Discussion

Insect hemolymph serves as a transit point connecting the primary infectious organ in the insect (the alimentary canal) to the terminal infectious tissue (salivary glands), making it critical for the systemic dissemination of persistent plant viruses in the bodies of their vectors (2, 35, 36). Viral propagation does not cause the cytopathology in the hemolymph, implying that the hemolymph employs the antiviral mechanism to limit viral excessive accumulation (37). However, viruses would evade or even exploit the antiviral immune pathway in the hemolymph of insect vectors to facilitate viral transmission.

PPO, a key protein in melanin biosynthesis during melanization, acts as the zymogen for the production of PO, which subsequently catalyzes the production of melanin (13–16). Various insect PPOs are produced by specific hemocytes (38). Indeed, our study reveals that PPO is primarily expressed in hemocytes cells of *R. dorsalis*. The cleavage of inactive PPO into active PO is essential for the synthesis of melanin. We demonstrate that the knockdown of *PPO* expression strongly reduces PO activity, which efficiently represses the melanization response in the hemolymph of *R. dorsalis*, highlighting the pivotal role of PPO in hemolymph-specific antiviral mechanisms in insects. We further determine that the plant rhabdovirus RSMV has evolved to evade the antiviral melanization response in the hemolymph, facilitating viral persistent propagation of insect vectors. After virions enter the hemolymph cells, viral protein N is initially synthesized and directly interacts with PPO, and this process would strongly activate the expression of PPO. However, the interaction of RSMV N with PPO could effectively inhibit the proteolytic cleavage of the zymogen PPO to active PO, finally suppressing the melanization and promoting viral infection. Similarly, the knockdown of PPO expression by RNAi or inhibitor treatment also suppresses the melanization and promotes viral infection, finally causing a high insect mortality rate. Thus, RSMV manipulates hemolymph melanization to maintain a delicate balance between viral effective propagation and persistent transmission, allowing for viral persistence and insect survival in nature.

Viruses have developed various mechanisms to counteract host melanization by targeting different steps involved in the melanin biosynthesis. For example, WSSV453, a virulence factor of white spot syndrome virus, interferes with melanization in shrimp by inhibiting the activity of the terminal serine proteinase PmPPAE2, which catalyzes the proteolytic of PPO into PO (24–26). Nucleopolyhedrovirus suppresses the melanization in *Helicoverpa armigera* larvae by inducing the expression of the serine protease inhibitors serpin-5 and serpin-9, which inhibit the PPO activity (27, 28). A rice tenuivirus suppresses melanization in planthopper vector by decreasing the cleavage ability of PPO (22). Thus, different viruses have developed the relative mechanisms to suppress melanization by decreasing the cleavage ability of PPOs. Interestingly, we find that the microinjection of purified N protein of RSMV into the body of leafhopper vector can effectively inhibit PPO activity and suppress melanization to facilitate viral infection. We thus speculate that N proteins of rhabdoviruses would serve as the effectors to recognize PPOs and inhibit their cleavage activities.

**Figure 7:**
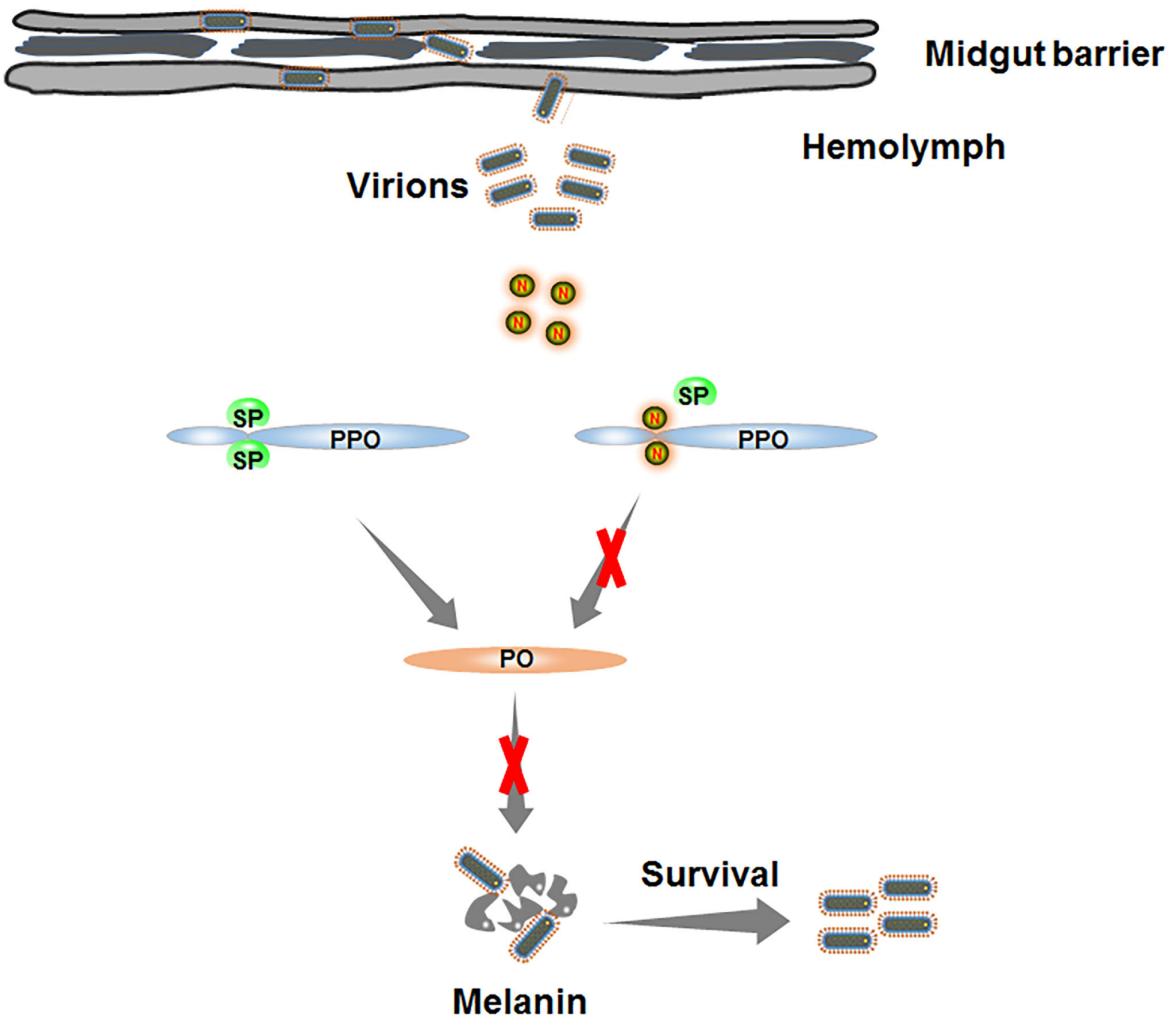
Schematic model depicting RSMV N-induced melanization evading in insect vectors. Once RSMV particles successfully overcome the midgut barrier and release into hemolymph. The serine protease (SP) is progressively activated to catalyze PPO into active PO. However, RMSV N directly interacts with PPO and prevents the production of PO, finally impairing the biosynthesis of melanin and benefiting the survival of RSMV from melanization.

Based on our findings, we propose a model depicting RSMV N-induced melanization evading in insect vectors (**Figure 7**). Once RSMV virions spread into hemolymph, viral propagation induces the melanization responses. The downstream serine protease cascade is progressively activated and catalyzes the proteolysis of PPO into active PO. PO, a key enzyme in melanin biosynthesis, mediates the production of melanin, which directly encapsulates and kills RSMV particles. To counteract melanization, RSMV N interacts with PPO and prevents its cleavage into active PO. Taken together, our findings reveal that RSMV has evolved to subvert the melanization-mediated antiviral defense in vector hemolymph *via* N-mediated inhibition of PPO cleavage to PO, finally facilitating viral persistent propagation in insect vectors. Our study expands our knowledge of how plant rhabdoviruses modulate melanization to ensure their propagation in insect vectors and enhances our understanding of the evolutionary trade-off between persistent plant viruses and insect vectors.

## Data Availability Statement

The raw data supporting the conclusions of this article will be made available by the authors, without undue reservation.

## Author Contributions

X-FZ and TW conceived and designed the experiments. RZ, X-FZ, YC, YX, and HC performed the experiments. RZ, X-FZ, and TW analyzed the data. X-FZ, ZG, and TW wrote the manuscript. All authors read and approved the final manuscript.

## Funding

This project was supported by funds from the National Natural Science Foundation of China (31871931) and the National Natural Science Foundation of China Major International (Regional) Joint Research Projects (31920103014).

## Conflict of Interest

The authors declare that the research was conducted in the absence of any commercial or financial relationships that could be construed as a potential conflict of interest.

## Publisher’s Note

All claims expressed in this article are solely those of the authors and do not necessarily represent those of their affiliated organizations, or those of the publisher, the editors and the reviewers. Any product that may be evaluated in this article, or claim that may be made by its manufacturer, is not guaranteed or endorsed by the publisher.
