# Supplementary material for: Nucleoprotein of a Rice Rhabdovirus Serves as the Effector to Attenuate Hemolymph Melanization and Facilitate Viral Persistent Propagation in its Leafhopper Vector
Source: Front Immunol. 2022 May 17;13:904244. doi: 10.3389/fimmu.2022.904244 (PMC9152149; doi:10.3389/fimmu.2022.904244)
Supplement: Supplementary file 1 [file Image_1.pdf]

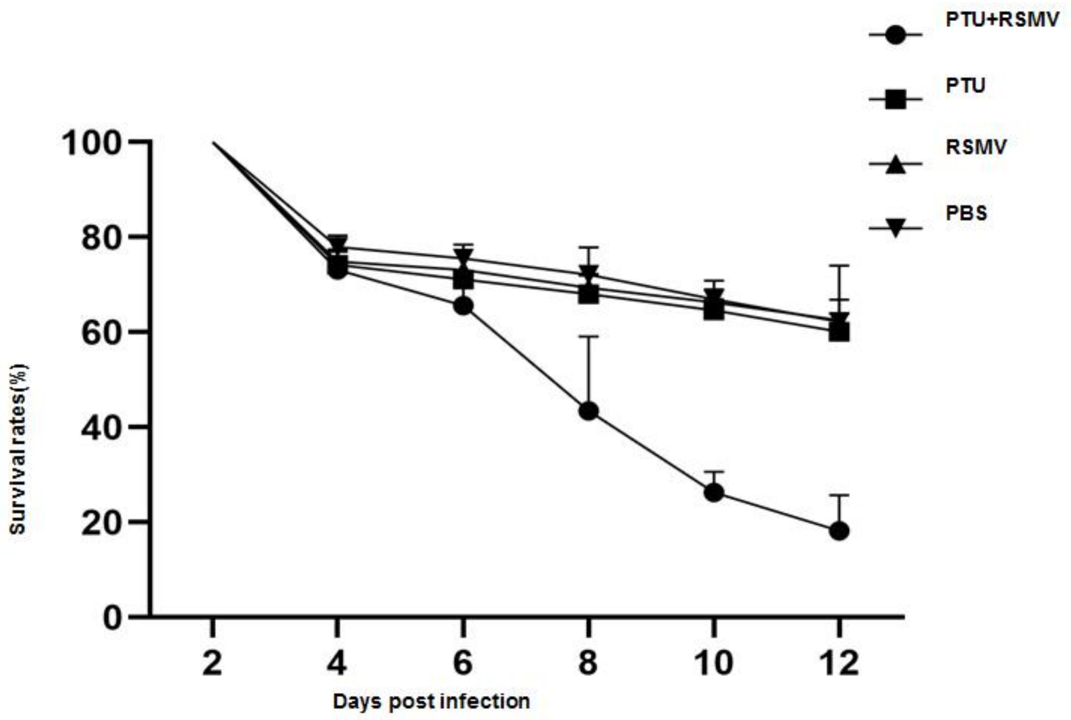

Supplementary Figure 1. The survival rates of viruliferous and nonviruliferous *R. dorsalis* following PTU microinjection.

Fifty viruliferous and 50 nonviruliferous *R. dorsalis* were microinjected with PTU (0.5 mM). The number of surviving *R. dorsalis* was counted at 2, 4, 6, 8, 10, and 12 d after treatment. Viruliferous or nonviruliferous *R. dorsalis* injected with PBS were used as controls. Each point represents the mean value of triplicates, and the error bars indicate standard deviation.
